# Adapting to an Ageing Health Workforce: Alternative Versus the Standard Ageing of Physicians and Nurses in Czechia

**DOI:** 10.1177/11786329261477828

**Published:** 2026-08-13

**Authors:** Filip Čábela, Tereza Patáková, Luděk Šídlo

**Affiliations:** 1 Department of Demography and Geodemography, Faculty of Science, 112302 Charles University, Prague, Czechia

**Keywords:** physicians, nurses, working life tables, alternative ageing indicators, health workforce planning, Czechia

## Abstract

**Background:**

This article examines the demographic ageing of physicians and nurses in Czechia between 2012 and 2022, a critical issue amid widespread healthcare professional shortages across Europe. The study applies standard and alternative indicators of demographic ageing to working life tables to identify trends and intensity in the ageing structure of the Czech healthcare workforce.

**Methods:**

Aimed at analysing the intensity of the ageing and attrition of healthcare professionals in the system, single-decrement working life tables were constructed employing individual anonymised data obtained from the largest Czech health insurance company, which has concluded contracts with practically all the country’s healthcare providers. Standard and alternative indicators of demographic ageing were subsequently applied to the working life tables in an adapted form (e.g. the constant prospective age, ageing index, etc.).

**Results:**

In addition to the most important finding that the healthcare workforce in Czechia is ageing, the results highlight that nearly one-third of female physicians in outpatient care (OC) had only 10 years of working life remaining in the period 2020–2022. A similar situation was observed concerning nurses across all three monitored intervals within the studied period. Overall, the healthcare system has failed to respond to the ageing of large older cohorts either by extending their professional careers or, more importantly, through generational renewal.

**Conclusions:**

Alternative aging indicators reveal that the demographic aging of the Czech health workforce is highly heterogeneous, with nurses posing a more critical threat than physicians due to their lower participation in the workforce after reaching retirement age. Standard workforce planning methods fail to capture these shifting retirement thresholds and dynamics between inpatient and outpatient care. To prevent personnel shortfalls, healthcare policymakers must transition from generic recruitment strategies to creating age-friendly working environments that prolong the professional careers of aging healthcare staff.

## Introduction

Demographic ageing is considered to be one of the most significant global phenomena of the 21st century and it is fundamentally transforming the structure of modern societies. Since the turn of the millennium, the number and proportion of individuals in older age groups have been on the increase in nearly every developed country worldwide.^1,2^ The prevalence and extent of demographic ageing result from the long-term interaction of three core processes: fertility, mortality and migration.^3^ The decline in fertility, often explained through the concept of the Second Demographic Transition,^4,5^ has led to so-called ‘ageing from the bottom’ (the narrowing of the base of the population pyramid).

This is accompanied by ‘ageing from the top’, which is associated with increasing life expectancy.^6^ However, it must be emphasised that a longer life does not automatically equate to an extension of healthy life years.^7,8^ This discrepancy, which is discussed frequently in the context of the expansion of morbidity theory,^9^ exerts enormous pressure on both social and healthcare systems. As noted by Bhattacharjee et al,^10^ by 2050, approximately one in six people worldwide will be over the age of 60, which will necessitate the redefinition of the labour market, the concept of lifelong learning and care provision models.^11^

The third component, migration, plays a role primarily in terms of replenishing the productive segment of the population and potentially temporarily retards the ageing process or, conversely, accelerates the process in regions of emigration.^12,13^ Although migration is the least stable component in this respect and remains very difficult to predict, it comprises an integral element of reproductive dynamics,^14^ and its significance in terms of population growth (or stagnation) is expected to increase going forward.

The impacts of demographic ageing are particularly pronounced in the healthcare sector, which faces so-called ‘double demographic pressure’. On the demand side, absolute and relative increases in the number of older adults is leading to rising demand for healthcare and a shift in its structure towards the treatment of chronic and multimorbid conditions.^15,16^ However, this trend is accompanied by ageing on the supply side – specifically, human resources in the healthcare sector.^17-19^

Almost one-third of physicians in OECD countries were aged 55 or older in 2023. This proportion even reached 40% in nine OECD countries. In Czechia, the share of those aged 55 and over stood at 39%. Although an absolute increase in the number of healthcare professionals – both physicians and nurses – was evident in Czechia between 2012 and 2022, the age structure of these professions has shifted significantly, with the centre of gravity moving towards older age groups.^20^ This shift is largely being driven by the cohort replacement effect.^12^ Large cohorts of physicians who entered the system in the 1980s are now reaching retirement age. Consequently, the absolute increase in numbers is being supported by the continued presence of these older professionals within the system, as well as, partially, by international migration rather than by a massive influx of younger generations that would effectively act to lower the average age. Hence, Czechia represents a valuable case study of a country that is facing the significant ageing of its healthcare workforce as a result of both its historical development and systemic inertia. The findings of this study thus potentially relate to other countries that risk critical healthcare worker shortages without the prospect of systemic changes.

The dynamics of workforce ageing are not homogeneous and vary by profession and the type of care provided. The primary care segment is the most vulnerable with respect to the medical profession in developed countries.^21^ In the Czech context, model projections by Šídlo et al,^22^ highlight that by 2035, up to 40% of general practitioner (GP) capacity and 55% of paediatrician capacity could be lost due to ageing and retirement unless adequate generational renewal is ensured.

The primary reasons for the insufficient entry of young physicians to the primary care sector include the long-standing lower attractiveness and prestige of the field compared to highly-specialised hospital-based medicine; consequently, this segment exhibits the highest proportion of physicians of senior ages.^23^ Although efforts to reform the educational curricula and strengthen primary care practice have intensified in recent years,^24^ the inertia of the healthcare education system remains a significant factor. The training of a fully qualified GP takes approximately ten years; therefore, changes in terms of educational policy are reflected in the age structure only with a significant time delay. The shortage of GPs is particularly critical since primary care has an important ‘gatekeeper’ function and is essential for the overall efficiency of the healthcare system.

The situation is even more acute and, in many respects, alarming with respect to non-physician professions, particularly nurses, who constitute the largest group of workers in the healthcare sector. This group is also suffering from a lack of adequate generational renewal due primarily to high turnover rates, premature departures from the profession and the declining interest in nursing studies in general.^25^ The decreasing interest in nursing and the reluctance to remain in the field are the result of demanding working conditions, low salaries and the high incidence of burnout syndrome.^26^ These factors were further compounded in recent years by the Covid-19 pandemic, which exacerbated stress levels and, in many countries, led to a wave of resignations.^27^

The traditional human resource analysis of the phenomena described above considers primarily the chronological age (e.g. the proportion of physicians aged 60+ or 65+). However, in the contexts of modern healthcare and increasing life expectancy, this approach is potentially misleading. As early as in the 1970s, Norman B. Ryder^28^ pointed out that as life expectancy increases, fixed thresholds of old age lose their significance. This concept was further developed by Sanderson and Scherbov,^1,29-31^ who introduced the concept of prospective age. Rather than measuring age by years lived (the retrospective view), this approach measures age by the number of years remaining to the end of life or career (the prospective view). The standard indicators provide a limited and partially skewed perspective on this issue, as they utilise a fixed old-age threshold determined by chronological age (e.g. 65 years). Consequently, they remain constant across time and space, failing to achieve a sufficiently objective comparison between populations with differing mortality patterns, which can ultimately lead to distorted conclusions.^1,29^ While standard indicators operate with a fixed threshold, alternative indicators employ a ‘sliding’ threshold that reflects improving mortality conditions.^15,32^ This approach, which views individuals through a dual-age lens (chronologically and prospectively), enhances the analytical value of the data and is increasingly being applied in the study of the medical sector.^33^ To assess how old a person (or a monitored population) is, two distinct perspectives on time are required: one looking into the past and the other into the future. Given that humanity is reaching increasingly higher ages (also in better health), a comparison of these two approaches is essential. Indeed, as Sanderson and Scherbov^29,30^ note in their papers, being 60 years old today is not the same as it was in the last century. This perspective is crucial concerning professional groups with high human capital, e.g. physicians, who often work significantly beyond the statutory retirement age. It allows for distinguishing between simple chronological ageing and the actual decline in labour potential.

The advantage of alternative (including prospective) indicators also lies in the fact that, beyond descriptive trends, they provide a more realistic estimation of dependency (by incorporating population shifts, they do not overestimate the ‘burden’) and account for sensitivity to external mortality shocks^15^ (which, concurrently, may represent a limitation of theirs – see the Limitations chapter). Their contribution to interpreting and forecasting changes within the healthcare system can be found in the refinement of predictive models for retirement (traditional models incorrectly assume a fixed retirement age of 65, whereas the actual retirement age for primary care physicians in the Czech Republic has historically hovered around 69).^34^ A further advantage may lie in their subsequent application at a regional level, as well as their utility for targeted regionalisation of care and education; in regions experiencing more rapid demographic ageing, healthcare provider networks can be optimised in cooperation with health insurance companies to address the growing demand.^15^

The aim of this study is to apply this innovative perspective to data on the Czech healthcare system. The study seeks to identify trends in the intensity of demographic ageing with concern to healthcare professionals in Czechia between 2014 and 2022. In this context, while the term ‘health workforce’ broadly encompasses both clinical (such as physicians, nurses, and midwives) and non-clinical personnel (including administrative, technical, and support staff),^35^ this study deliberately narrows its focus to physicians and nurses. This selective scoping is justified by the fact that these two professions constitute the absolute core of the clinical health workforce and are directly responsible for the delivery, quality, and accessibility of primary, specialized outpatient, and inpatient care. Moreover, as both professions require extensive, highly specialized educational pipelines and long-term training, they exhibit a high degree of systemic inertia. Consequently, personnel shortages driven by the demographic aging of physicians and nurses pose the most immediate and critical threat to the overall resilience and sustainability of the healthcare system.

The analysis of working life tables relied on both standard (chronological) ageing indicators and their alternative counterparts – prospective indicators. Further objectives included the determination of whether physicians and nurses are affected by ageing differently and the roles played by gender and employment in outpatient (OC) versus inpatient (IC) care. One of the key research questions concerned whether the healthcare workforce is ageing more rapidly than the shift in the threshold for their departure from the profession, i.e. whether the system is able to adapt via ‘prospective rejuvenation’ by the extension of careers, or whether it faces a real risk of personnel collapse due to potential labour exhaustion. The answer to this question is central to the development of sustainable human resource management strategies and the capacity planning of the education system.

## Data and Methodology

Two primary registries of healthcare professionals are maintained in Czechia, both of which are managed by the Institute of Health Information and Statistics of the Czech Republic (IHIS CR). However, they are not particularly suitable for the monitoring of trends due to the absence or incompleteness of historical data. Hence, this study employed anonymised individual-level data on physicians and nurses provided by the General Health Insurance Company of the Czech Republic (GHIC) (see the Statements and Declarations section). The GHIC is the largest health insurance fund in Czechia and has concluded contracts with practically all the country’s healthcare providers, thus ensuring access to an almost complete dataset of active professionals.

The dataset consisted of data for the period 2012–2022 (as of 31st December), disaggregated by the provider type, gender and age. The provider type was further aggregated into two main categories: inpatient care (IC) and outpatient care (OC).

While the baseline GHIC registry contains records of contracted weekly working hours, this study strictly operates with individual headcounts to evaluate the intensity of workforce ageing. For the purposes of this analysis, data were aggregated by physical presence at the total workforce level as well as across both sectors (OC and IC). Consequently, part-time workers are included as distinct, equivalent active units (headcounts) in the life table models. The methodological decision to utilize headcounts rather than Full-Time Equivalents (FTE), along with its potential to overestimate the actual working capacity of the oldest age groups, is explicitly addressed and discussed in the Limitations section.

To ensure the homogeneity, statistical reliability, and reproducibility of the analysis, strict inclusion and exclusion criteria were applied to the initial dataset:

Inclusion Criteria:

• Active Professional Status: Only physicians and nurses who were actively practicing and held a valid contract with the GHIC as of December 31st of each monitored year were included in the analysis.
• Professional Qualifications (Nurses): The nursing workforce was strictly limited to individuals who had attained at least a secondary education completed with a school-leaving certificate, corresponding to fully qualified nursing staff.
• Sector Categorization: Subjects had to be clearly classifiable into one of the two core healthcare sectors: inpatient care (IC) or outpatient care (OC).

Exclusion Criteria:

• Male Nurses: Male individuals within the nursing profession were excluded from the analysis. This exclusion was necessary due to the extremely low proportion of males in the Czech nursing workforce (accounting for less than 5% in IC and less than 2% in OC, as detailed in Figure 3).
• Out-of-System Providers: Healthcare professionals operating exclusively outside the public health insurance system (e.g., purely private cosmetic surgery clinics or non-participating cash-only practices) were excluded, as they are not tracked within the GHIC registry. Given the institutional setting of the Czech healthcare system, the share of healthcare providers operating entirely outside this public framework is negligible. Consequently, their exclusion does not introduce any systematic bias and the dataset can be considered highly representative of the nation’s actual health workforce capacity.

### Working Life Tables

Single-decrement working life tables were constructed for the analysis of the intensity of ageing and the attrition of healthcare professionals in the system. Working life tables comprise one of the fundamental tools of demographic analysis, primarily in the field of mortality.^36^ The core element for constructing working life tables concerns the calculation of exit probabilities. The methodological procedure for calculating the probabilities of exit ( $q_{x}$ ) and their subsequent statistical smoothing was based on the methodology applied and described in detail in a previous study that focused on primary care physicians.^34^

The model considers two states: remaining active within a given care sector and exiting the system (due to retirement, death, career change, migration, etc.). The probabilities of exit were calculated applying the direct method based on the cohort-based tracking of the presence of individual professionals in the dataset between successive years:

qxx+1g,ptc=Oxx+1g,ptcPxg,p31.12.t−1c

where qxx+1g,ptc is the probability of exit from the system during year t between ages x and x+1, Oxx+1g,ptc represents the number of the HWF who exit the system in year t and Pxg,p31.12.t−1c is the number of HWF at the end of year t−1 . C is the generation (the year of birth) of the HWF, g is the gender and p is the type of health care provider.

Specifically, an exit from the healthcare system is operationally defined as the complete absence of a professional’s unique identification number (ID) from the GHIC database in the subsequent year ( t+1 ). A major data limitation is that the registry does not record the specific reasons for this attrition. Therefore, the model cannot differentiate whether a professional exited the workforce due to standard statutory retirement, mortality, emigration abroad, or premature career abandonment caused by burnout or a change in profession.

In accordance with a previously published methodology,^34^ the probabilities of exit were calculated for individual years, then averaged and subsequently smoothed applying two consecutive methods. A 9-period weighted moving average (WMA 9) was applied for the younger age groups (with a sufficient number of observations). With regard to the oldest age groups, concerning which the empirical data exhibit higher fluctuations, the Gompertz-Makeham function was applied as estimated via the King-Hardy method (the suitability of the parameters was verified applying the Akaike Information Criterion). The maximum age (where the probability of exit equals 1) was set in the model at 90 years for physicians and 80 years for nurses.

Tables were constructed based on the exit probabilities, the primary output of which comprised the working life expectancy (WLE), which was defined as the expected average number of remaining years of working life at an exact age.^37^ In order to examine the changes that occurred in terms of the dynamics of healthcare workforce ageing over time in greater detail, the monitored period was divided into three triennial intervals: 2014–2016, 2017–2019 and 2020–2022. The baseline period of 2012–2022 was determined by the historical availability of consistent individual-level data from the GHIC registries, aiming to capture a stable decade of development. The subsequent pooling of this timeframe into three triennial sub-periods served two methodological purposes. First, it effectively smoothed out random, minor annual fluctuations in exit behaviors, thereby stabilizing the calculation of empirical exit probabilities for lower-frequency cohorts. Second, this triennial structure allowed us to isolate the 2020–2022 period, capturing potential structural anomalies in workforce retention caused by the COVID-19 pandemic without distorting the baseline trends of the preceding years.

### Ageing Indicators

As part of the research, standard and alternative approaches to measuring demographic ageing were applied to the populations of physicians and nurses – specifically to the working life tables and the associated working life expectancy. Two indicators were required for the basic calculation of the level of demographic ageing of the healthcare professionals: the number of healthcare professionals by age $\left(P_{x}\right)$ and their working life expectancy ( $e_{x}$ ), as derived from the working life tables.

The determination of the prospective age represented the most important foundation for the calculation of the alternative indicators of demographic ageing. The prospective age denotes ‘*the age assigned to a given population in a given year based on the same remaining life expectancy in a reference year (and population)*’.^33(p131)^ According to Sanderson and Scherbov,^38^ the prospective age can be defined as the average number of years of life remaining for a given person or group of persons – specifically, the number of years that remain until the probable age of death under the prevailing mortality conditions. Applying the linear interpolation method, the precise prospective age can be estimated according to the following formula^39(p20)^:

$$x=x_{0}+\left(z-z_{0}\right)\frac{x_{1}-x_{0}\;}{z_{1}-z_{0}\;}$$

where x denotes the age and z represents the remaining working life expectancy at age x . $x_{0}$ subsequently identifies the age at which the remaining working life expectancy is still higher than the value we wish to determine, while $x_{1}$ denotes the age (higher than $x_{0}$ ) at which the working life expectancy is already lower than the determined value. Regarding the variable z (the remaining working life expectancy of the sought prospective age), $z_{0}$ denotes the remaining life expectancy at age $x_{0}$ , and $z_{1}$ denotes the remaining working life expectancy at age $x_{1}$ .

Following discussions between the authors, the so-called **constant prospective age** with a duration of 10 years (***CPA RWLE10–***) was selected for analytical purposes. This age is based on the principle that we aim to determine the age at which the remaining life expectancy (in this case, the remaining working life expectancy) is equal to 10 years for each population in every given year, for which the linear interpolation method can be employed. Further indicators related to demographic ageing were computed following the calculation of the constant prospective age values for physicians and nurses, i.e. the proportions of older persons and the ageing indices.

The indicator that directly followed the calculation of the constant prospective age was referred to as **the proportion of persons with a remaining working life expectancy of 10 years or less (*Prop. RWLE 10–*)**. Here, the criterion of 10 remaining working years serves as the equivalent to the standard indicator, **the proportion of persons aged 65 and over (*Prop. 65+*)**, while simultaneously establishing an alternative old-age threshold to the traditional 65 years (the numerator is the number of persons with 10 or fewer years of remaining working life, or those aged 65 and older, respectively). Both proportions are then related to the total population of physicians or nurses (by gender). The calculation formulae are as follows:

$$\begin{array}{ll} Prop.\;RWLE\;10-=\frac{P_{x_{RWLE10-}}}{P}*100 & Prop.\;65+=\frac{P_{65+}}{P}*100 \end{array}$$

Ageing indices were employed as additional indicators in the analysis. The standard **ageing index (*AI*)** is calculated as the ratio of the number of persons aged 65 and over to the sum of persons aged 39 and under. The age group 39 and under was determined as the equivalent to the 0–19 age category in terms of representing the young age component. This age was selected primarily since the 0–19 age category is currently considered pre-productive, i.e. young people typically enter the workforce from the age of 20 onwards. Thus, for the purposes of the study, the age of 39 corresponded to the aforementioned 19-year span. This approach maintained the same interval for the younger age category as that used in calculations of general population demographic ageing. The alternative counterpart comprises **the prospective ageing index (*PAI*)**, which is logically calculated as the ratio of the oldest working age category (the number of persons with a remaining working life expectancy of 10 years or less) to the sum of persons under 39. The formulae for the two indices are as follows:

$$\begin{array}{ll} PAI=\frac{P_{x_{RWLE10-}}}{P_{39-}}*100 & AI=\frac{P_{65+}}{P_{39-}}*100 \end{array}$$

The final two indicators comprised the standard old-age dependency ratio and its alternative counterpart the prospective old-age dependency ratio. The standard **old-age dependency ratio (*OADR*)** is the result of the ratio of the number of persons aged 65 and over to the number of physicians/nurses in the 40–64 age category. **The prospective old-age dependency ratio (*POADR*)** is calculated applying the same principle; however, the number of persons in the age category with a remaining working life expectancy of 10 years or less is used as the numerator, while the denominator refers to the sum of persons aged 40 up to the prospective age. The formulae are as follows:

$$\begin{array}{ll} POADR=\frac{P_{x_{RWLE10-}}}{P_{{40-x}_{RWLE>10}}}*100 & OADR=\frac{P_{65+}}{P_{40-64}}*100 \end{array}$$

It is important to note that the linear interpolation method was employed in all the calculations of the alternative indicators, as explained by the fact that if, for example, e_55_=10.40 and e_56_=9.57, then the group of persons with a remaining working life expectancy of 10 years or less includes both those aged 56 and over and the corresponding proportion of 55-year-olds. Other indicators and alternatives, e.g. the prospective median age, could also be used to calculate the level of ageing in the healthcare population; however, they were not included in the scope of this study.

## Results

The analysis of the working life tables for healthcare professionals revealed a significant difference in terms of the ageing dynamics across all the monitored aspects – both by profession and type of provider. While standard ageing (the increase in the average age) is evident across the entire sector, the alternative indicators revealed varying capacities for adaptation for the individual segments. Consequently, demographic ageing differed between physicians and nurses, as well as the type of care provision (inpatient/outpatient). The article continues firstly with the analysis of physicians, followed by that of nurses.

### Physicians

**Figure 1.**
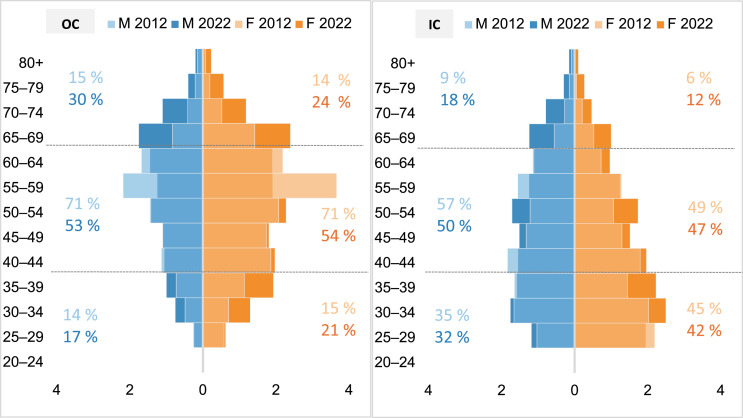
Number of physicians by age, gender and type of care provider (in thousands). Source: Data from GHIC CR; author’s own calculations. *Note.* The horizontal lines divide the population into three main age cohorts: <40, 40–64 and 65+. The floating percentage values represent the aggregated share of each specific age cohort in the total workforce for 2012 (lighter) and 2022 (darker)

The analysis of the age structure serves as the starting point for understanding the dynamics of demographic ageing. Between 2012 and 2022, a significant transformation occurred in the age structure of physicians with respect to both the inpatient care (IC) and outpatient care (OC) settings. As revealed by the population pyramid (Figure 1), the proportion of physicians in the 65 and over age group doubled during this period. However, a fundamental structural difference is evident between the bases of the IC and OC pyramids. While the IC pyramid features a strong base of young physicians, the OC pyramid exhibits a regressive shape. This disparity was driven by the natural career cycle: physicians typically begin their careers in IC facilities in order to complete their specialist training and subsequently a portion of them transitions to the OC sector.

**Figure 2.**
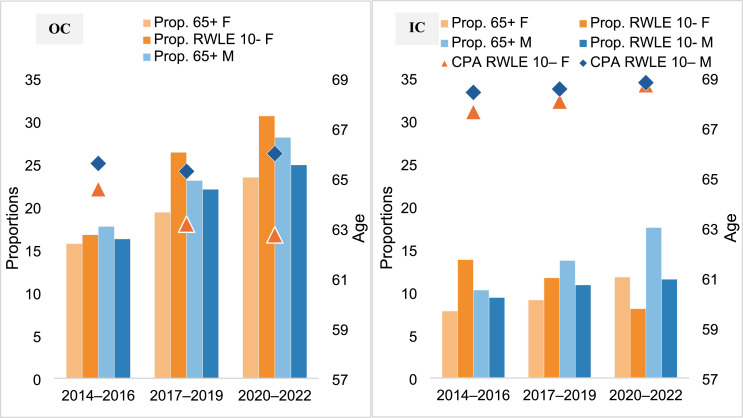
Trends in the standard vs. prospective ageing indicators for physicians by type of care provider (2014–2022). Source: Data from GHIC CR; author’s own calculations

The demographic ageing analysis revealed that the most critical situation concerned OC female physicians. Although this group experienced significant chronological ageing (an increase in the proportion of those aged 65+), no adequate career extension or generational renewal was evident (Figure 2). The prospective age declined over time from 64.5 to 62.7 years. As a result, nearly one-third (30.6%) of female OC physicians had only 10 years of working life remaining (RLE 10–) in the 2020–2022 period. This was further inferred from the age structure (Figure 1), concerning which a sharp decline in the number of female OC physicians occurred between 2014 and 2022 within the 55–64 age group (6 016 physicians) and the 65–74 age group (3 582 physicians), as well as from increases in the values of both the prospective ageing index and the prospective old-age dependency ratio (Table 1).

**Table 1.** Trends in the Standard vs. Prospective Ageing Index and Old-Age Dependency Ratio for Physicians by Type of Care Provider (2014–2022)

Index	Gender	OC (Providers of outpatient care)			IC (Providers of inpatient care)		
		2014–2016	2017–2019	2020–2022	2014–2016	2017–2019	2020–2022
AI	F	124.0	127.6	133.8	22.4	25.7	34.2
	M	145.7	172.0	191.7	37.0	52.3	69.0
PAI	F	132.3	173.7	174.6	39.7	33.0	23.5
	M	133.7	164.4	169.9	33.9	41.4	45.3
OADR	F	21.9	29.6	39.7	13.6	16.4	21.8
	M	25.3	36.3	49.1	16.5	22.8	30.7
POADR	F	23.7	45.1	59.0	26.8	22.1	14.0
	M	22.7	34.2	41.2	14.9	17.2	18.2

Source: Data from GHIC CR; author’s own calculations

Although the proportion of female physicians aged 65 and over increased with respect to both IC and OC, the system underwent ‘alternative rejuvenation’ during the studied period. Female physicians in hospitals deferred the threshold for exiting the profession (the prospective age increased from approximately 61 to 67 years), which resulted in a decline in the proportion of those with 10 years of working life remaining from 13% to 8.1% (Figure 2). This positive mechanism was supported by the entry of younger and ‘middle-aged’ female physicians into the system (Figure 1), which was reflected in decreases in both the prospective ageing and dependency indices (Table 1). Consequently, the hospital sector was demographically more stable than the OC sector concerning female physicians at the end of the studied period.

The ageing dynamics of male physicians were strongly influenced by the dwindling entry of the younger generation to the profession. Significantly fewer young male physicians are currently entering the system than their female counterparts, which is resulting in the masculinisation of the older age and the feminisation of the younger age categories (Figure 1).

With respect to OC physicians, we observed that even a high prospective age (and its slight increase over time) is insufficient to compensate for the ageing of large cohorts. Both the prospective ageing index and the prospective old-age dependency ratio increased during the studied period, although not as sharply as the standard indices (Table 1). Alternatively, the population of OC physicians aged more slowly; however, even though these physicians are extending their professional careers, the prospective proportion is increasing. Consequently, one-quarter of male physicians had only 10 years of working life remaining in the period 2020–2022 (Figure 2).

Although IC physicians extended their careers by nearly 3 years during the monitored period (Figure 2), the larger cohorts shifted towards older age groups. Alternatively, this group is younger; just 11.5% of physicians had 10 years of working life remaining in the final monitored period of 2020–2022. However, the fact remains that this population is also ageing. The system currently benefits from the fact that a large number of IC physicians remain in the system even after reaching retirement age – particularly the 65–74 age group in 2022. Nevertheless, this represents a partial and long-term unsustainable substitute. IC physicians are thus effectively working beyond their standard tenure, while the numbers of younger physicians in the system are insufficient to fully compensate for the future exit of their older counterparts.

The analysis revealed a decline in the number of males at both younger and mid-career ages. Coupled with the shift of the larger cohorts towards older age groups, this provides a clear example of demographic ageing (Table 1), which will be difficult to reverse.

### Nurses

**Figure 3.**
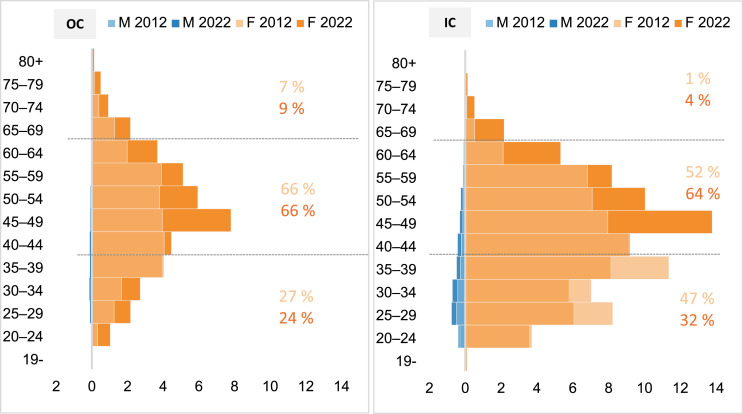
Number of nurses by age, gender and type of care provider (in thousands). Source: Data from GHIC CR; author’s own calculations. *Note.* The horizontal lines divide the nursing population into three main age cohorts: <40, 40–64, and 65+. The floating percentage values represent the aggregated share of each specific age cohort in the total workforce for 2012 (lighter) and 2022 (darker)

The age structure of nurses revealed a structurally differing working life pattern than that of physicians. As shown in Figure 3, nursing remains a heavily feminised profession (women account for 95% of IC and 98% of OC nursing personnel); therefore, the following ageing analysis focused exclusively on the female nursing population. A further significant difference lies in the fact that, compared to physicians, fewer nurses work at older ages. In other words, their career paths end significantly earlier: the average age of exiting the profession ranges from 61 to 64 years (upon reaching retirement age).

With regard to the change in the age structure between 2012 and 2022, a noticeable increase in the number of OC nurses is evident across all the age cohorts. In contrast, increases were evident for IC nurses only in those age groups over 45, with a corresponding decline in the number of nurses in the younger age categories.

**Figure 4.**
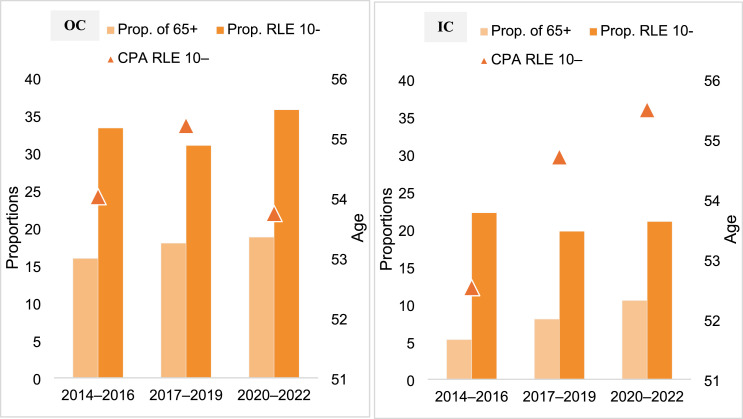
Trends in the standard vs. alternative ageing indicators for nurses by type of care provider (2014–2022). Source: Data from GHIC CR; author’s own calculations

The situation concerning nurses employed by OC providers is the most critical of all the analysed groups, as shown in Figure 4. Chronological ageing in this sector is progressing particularly rapidly; however, unlike physicians, this trend is not being compensated for by the extension of the careers of nurses. The prospective old-age threshold (the age at which a nurse has, on average, 10 years of working life remaining, i.e. RLE 10–) is almost stagnant and no shift towards older ages is evident. As a consequence, one-third of OC nurses had only 10 years of work remaining during the 2014–2022 period. This relatively critical situation can be attributed to a combination of the ageing of the larger nursing cohorts and the shortening of working life expectancy. Unlike physicians, a significant proportion of nurses tend to lack both the inclination and the physical and/or mental capacity to “work beyond their tenure”. The system is failing to adapt and is approaching its limits in terms of personnel capacity.

At first glance, the prospective indicators for the IC sector revealed a surprising paradox. While both the absolute numbers and the chronological ages of hospital-based nurses increased, the proportion of IC nurses with a remaining working life expectancy of 10 years or less declined slightly in the studied period. From the perspective of this indicator, it appears that the hospital sector has undergone ‘alternative rejuvenation’. However, the slight decline was due more to a minor shift in the departure threshold and the survival effect of the largest cohorts in mid-career rather than by a genuine improvement in the situation. Other prospective indicators, however, appear to negate such optimism. Both the prospective ageing index (PAI) and the standard ageing index (AI) are increasing in the IC sector (Table 2). Thus, it is evident that while older nurses are remaining in the system for slightly longer than previously, fewer younger persons are entering the profession (the ageing index denominator is decreasing).

**Table 2.** Trends in the Standard vs. Prospective Ageing Index and Old Age Dependency Ratio for Nurses by Type of Care Provider (2014–2022)

Index	OC (Providers of outpatient care)			IC (Providers of inpatient care)		
	2014–2016	2017–2019	2020–2022	2014–2016	2017–2019	2020–2022
AI	68.3	90.5	90.9	12.9	23.7	35.2
PAI	142.9	156.2	173.2	53.9	58.2	70.4
OADR	26.2	28.9	30.9	9.9	13.8	17.6
POADR	76.7	63.0	81.9	60.6	42.5	42.9

Source: Data from GHIC CR; author’s own calculations

Moreover, it was observed that the prospective old-age dependency ratio (POADR) for the IC sector is declining (Table 2), which signifies that the centre of gravity of the workforce is concentrated in the middle-age groups (40–60 years). Mid-career nurses, who are currently sustaining hospital staffing levels, have not yet reached the prospective old-age threshold (RLE 10–). However, as soon as this large cohort shifts beyond 60 years of age during the following decade, the IC sector will face a massive and sudden shortfall in personnel.

## Discussion

The results of our study clearly demonstrate differences in terms of the ageing dynamics of the analysed professions. The research identified that while physicians are ‘ageing within the system’ (working to higher ages), nurses are tending to exit the system at lower ages. This phenomenon closely corresponds with the findings of other related studies,^22,40^ which confirmed that the ageing of nursing staff is becoming a profound structural problem. Nurses are leaving the healthcare system significantly earlier than physicians, a factor that has often been attributed to the high physical and psychosocial demands of the nursing profession.^41,42^ At the same time, nurses face lower baseline remuneration and reduced professional recognition compared to physicians.^43^ Current indicators suggest that student interest in nursing programs is steadily declining across OECD countries, a trend further exacerbated by the COVID-19 pandemic, during which a massive increase in workload and subsequent burnout occurred.^26,44^

As You and Donnelly^45^ point out, population ageing, coupled with a high demand for healthcare services, is presenting a challenge for systems that – given the simultaneous ageing of nurses – appears likely to lead to critical personnel shortfalls. Although the average age of physicians is increasing, their ability and willingness to remain in the system longer is partially mitigating personnel deficits. However, as Buerhaus et al,^46^ point out, this development is accompanied by risks associated with physical limits and operational strain.

One of the key themes of our analysis concerned the capacity of the system to adapt via the extension of working life. However, the willingness of healthcare professionals to remain in employment for longer periods is contingent upon a range of factors. Studies by Sun et al,^47^ and de Vries et al,^48^ both emphasised that the decision to defer retirement is primarily determined by job satisfaction, levels of occupational fatigue and the ability to achieve the ideal work-life balance. Conversely, complex organisational environments and high demands potentially increase the risk of departure. Ageism comprises one of the barriers that influences the retention of older workers. Although the literature frequently focuses on discrimination against older patients,^49^ ageism also manifests itself in relationships between healthcare professionals. As Pritchett and Kinsley^42^ revealed, negative stereotypes in the nursing environment have the potential to exert a devastating impact on the mental health of older nurses and accelerate their decision to leave the profession. The provision of support and recognition by management is therefore crucial for the retention of experienced staff.

The evaluation of the results of our study further included the consideration of the broader macro-demographic context. Changes in the age structure of Czech healthcare professionals are influenced significantly by the cohort replacement effect.^12^ As our data suggest, the system is currently experiencing the transition of larger cohorts towards older age groups. Consequently, the quantification of expected changes in the age structure is essential in terms of the timely identification of potential social, economic and healthcare challenges.^32^ Furthermore, the situation has been complicated by external shocks in recent years. The Covid-19 pandemic revealed the vulnerability of healthcare systems that are dependent on older physicians, for whom the disease posed a high health risk.^46^ Alternatively, several developed countries see migration as a potential approach to attaining labour market stabilisation.^13^ Migration plays a significant role in terms of replenishing the productive age group of the population and rejuvenating the labour market.^50,51^ However, given its inherent instability, migration should not be seen as the sole universal panacea for generational renewal.

This study demonstrated the limitations of the traditional concept of demographic ageing based solely on chronological age. The reason for applying alternative indicators concerned an effort to enhance the explanatory power of the data at our disposal. As demonstrated by Havelková and Šídlo^34^ and Čábela and Šídlo,^32^ linking demographic indicators with actual employment and remaining working life expectancy provides a far more accurate picture of the real depletion of human capital than the consideration of the chronological age alone. The prospective approach proves that ageing should not be perceived in purely negative terms; the identification of shifting old-age thresholds opens up new opportunities in terms of active ageing and the participation of seniors in the labour market.^52-54^

To fully understand the analytical value of this dual-age lens, it is essential to contrast the findings yielded by standard versus prospective indicators, which reveal critical micro-dynamics that remain entirely hidden in purely chronological assessments. A prime example of this added value can be observed among female physicians in IC sector. According to the standard Aging Index ( AI ), this population is experiencing rapid, alarming aging, with the index values jumping from 22.4 to 34.2 over the monitored period. In a traditional workforce planning framework, this trend would likely be misinterpreted as a signal of imminent staff depletion, potentially generating undue concern among hospital administrators. However, our alternative Prospective Aging Index ( PAI ) paints a completely different picture, actually decreasing from 39.7 to 23.5. This statistical divergence odcovers a vital mechanism of systemic adaptation: while these female hospital physicians are chronologically older, they are actively extending their professional careers and successfully deferring their departure from the workforce. The system is thus undergoing a form of “alternative rejuvenation” through career extension, a positive and stabilizing trend that traditional chronological data completely obscure.

Conversely, the prospective indicators serve as a crucial early-warning system in sectors where apparent chronological stability masks underlying structural vulnerabilities. In the IC nursing sector, the Prospective Old-Age Dependency Ratio ( POADR ) exhibited a notable decline from 60.6 to 42.9. While a superficial reading of this indicator might suggest an improvement in workforce sustainability, the simultaneous increase in both the standard ( AI ) and prospective ( PAI ) aging indices expose a more dangerous underlying reality. The center of gravity of the hospital nursing workforce has become highly concentrated in the mid-career cohorts (aged 40–60), who have not yet crossed the remaining working life threshold of 10 years ( RLE 10− ). This implies that the current stability of hospital staffing levels relies almost entirely on a single generation of experienced nurses. Rather than indicating long-term health, this prospective compression warns policymakers that as this massive cohort moves past the age of 60 over the next decade, the IC sector will face a sudden, massive wave of concurrent retirements that the dwindling influx of younger generations will be entirely unable to replace.

In the context of the Czech healthcare system, these dynamic ageing trends are deeply intertwined with specific institutional practices and historic structural imbalances. The critical aging observed among outpatient care (OC) physicians, where nearly one-third of females are within 10 years of retirement, reflects long-standing systemic incentives. In Czechia, most physicians naturally begin their careers in hospital-based IC facilities to complete their compulsory post-graduate specialty training. A significant portion subsequently transitions to the OC sector later in life, drawn by more predictable working hours and a lack of mandatory overnight shifts. However, this historical career path has resulted in a severe generational bottleneck in primary and outpatient care. Furthermore, the persistent gender pay gap in the healthcare sector, combined with the extreme physical and psychological toll of hospital night shifts, heavily influences the career trajectories of female physicians, often forcing an earlier reduction in workload or a transition to the outpatient sector.^55,56^

These prospective findings have direct, urgent implications for workforce planning, educational pipelines, and migration policies. Because the training of a fully qualified independent physician in Czechia requires an educational pipeline of approximately ten years, the fact that 30.6% of female OC physicians are entering their final decade of practice means that the capacities of medical faculties and outpatient residency programs should have been expanded years ago. Relying purely on historical headcount averages for capacity planning is no longer sustainable. For the nursing profession, where the lower degree of “over-tenure” stems from sheer physical and mental exhaustion, workforce planning cannot simply revolve around recruitment targets. The educational pipeline for nurses is already facing a severe shortage of applicants; therefore, immigration pathways must be streamlined specifically for non-physician staff. More importantly, practical policy must focus on creating an aging-friendly workplace culture–such as introducing mandatory physical therapy, restricting consecutive night shifts for older staff, and establishing phased retirement plans–to actively prolong the career lifespans of the existing workforce.

The professional literature concurs that while the ageing of the healthcare workforce cannot be fully eliminated, it can be effectively managed. Our findings highlighted the necessity of implementing targeted strategies aimed at retaining workers in the healthcare system. Recommended measures include the introduction of flexible working arrangements, part-time contracts and phased retirement plans.^47,48^ A further option concerns the adaptation of professional roles – experienced older physicians and nurses could serve as mentors or clinical leads, thereby reducing their physical workload^41^ while preventing the loss of institutional memory. At the systemic level, it is essential that personnel planning be closely linked with demographic projections,^22^ otherwise the system will face the genuine risk of deterioration in terms of both its quality and accessibility.

### Limitations and Suggestions for Future Research

Several methodological and data limitations must be considered when interpreting the results of this study. First, although the individual-level data obtained from the General Health Insurance Company (GHIC) ensure an exceptionally high degree of representativeness due to the universal nature of the Czech public health insurance system, they do not include information on the negligible segment of healthcare professionals who operate entirely outside this public framework. Furthermore, the database does not record specific reasons for exiting the system. Operationally, an exit is identified solely by the complete absence of a professional’s unique identification number (ID) in the registry in the subsequent year. Consequently, our model is deterministic and single-decrement; it does not distinguish whether a professional exited the workforce due to standard statutory retirement, mortality, permanent emigration abroad, or premature career abandonment caused by burnout or a change in profession. This lack of granular data on attrition reasons limits our ability to evaluate the precise weight of socio-economic versus biological factors in workforce depletion.

Furthermore, aimed at maintaining the robustness of the data and ensuring a sufficient number of cases in the older age groups, we opted to aggregate healthcare providers in the form of just two categories: inpatient (IC) and outpatient (OC) care. This approach potentially masks significant differences between specific specialisations (e.g. between acute and long-term care, with potentially differing turnover rates). Also aimed at ensuring statistical reliability, males were excluded from the nursing analysis; hence, the results reflect exclusively the female nursing population and cannot be fully generalised to cover the entire profession.

A further limitation concerned the use of single-decrement life tables, which did not allow for the dynamic modelling of transitions between the IC and OC sectors. Therefore, the analysis of inter-sectoral mobility applying multi-state models represents a significant opportunity for future research in this field.

Certain limitations must also be recognised within the prospective indicators of the alternative approach. Although healthcare professionals have a certain number of years remaining to be worked, it is not entirely clear whether this will imply the same level of productivity, which may lead to an overestimation of the workforce. Related to this is the fact that retirement can be influenced by a whole range of factors – such as health status, organisational conditions, family reasons, workload, etc. – which can naturally affect the accuracy of the estimated remaining years of working life. Furthermore, a conceptual risk of the prospective age approach must be acknowledged: by defining the value of a workforce through the lens of remaining career years, it could inadvertently devalue older professionals due to their shorter future contribution. It is vital to state that alternative indicators measure the quantitative time horizon of the system’s capacity, not the qualitative and institutional value of experienced senior staff. A further limitation can be observed in the high sensitivity to external shocks, the impact of which on prospective indicators is evident.

Finally, the fact that the analysis primarily works with headcounts rather than full-time equivalents (FTE) represents an additional limitation. While the GHIC registry tracks weekly contracted working hours, allowing for the conversion of individual capacities into FTEs at an aggregated level, the primary visual models and standard indicators applied in this study rely on physical headcounts. The consideration of the actual volume of hours worked is an important factor in the context of demographic ageing since older healthcare professionals (particularly in the OC sector and those that remain in the profession beyond retirement age) often gradually reduce their working hours and clinical workload. By treating every active professional as a single unit regardless of their true contract volume, our headcount-based model potentially overestimates the actual operational capacity and future labour supply of the oldest age groups. Incorporating the development of working hours and actual time worked into the calculation of prospective ageing indicators represents a further opportunity for follow-up research.

## Conclusion

Nearly one-third (30.6%) of female physicians in the OC sector had only 10 years of working life remaining in the period 2020–2022. The healthcare system has failed to respond to the ageing of older, larger cohorts via the extension of working life, and even less so through generational turnover. The proportion of female IC physicians with 10 or fewer years of working life remaining declined from 13% to 8% during the studied period due primarily to the entry of younger and mid-career female physicians to the system. With respect to male physicians in the OC sector, even a high prospective age and its slight increase in later years were insufficient to offset the ageing of the population. Going forward, this factor has the potential to lead to an even greater shortage of male OC physicians in the system. Our analysis revealed that male IC physicians have a higher prospective age than their OC counterparts and that they extended their careers by approximately three years during the studied period.

In summary, it can be stated that female physicians are older in standard terms, whereas male physicians are older in alternative terms. Furthermore, the ageing indices for physicians are higher for OC providers than for IC providers, which is in line with the fact that the majority of physicians begin their careers in the IC sector and transition to the OC sector at later ages.

The situation of OC nurses is just as precarious as that of female physicians in the same healthcare setting. Approximately one-third of OC nurses had 10 or fewer years of working life remaining in all three of the monitored sub-periods (33% in 2014–2016; 31% in 2017–2019; and 36% in 2020–2022). Furthermore, nurses in both the OC and IC sectors are ageing more rapidly in alternative terms than in standard terms. The demographic ageing of nurses represents a more significant threat to the healthcare system than that of physicians due to their lower degree of “over-tenure”, i.e. working beyond retirement age. From the healthcare policy perspective, it is therefore essential that the system focuses on introducing systematic adjustments to working conditions and the job content of older nurses (e.g. reducing the physical workload) so as to ensure that they are both able and willing to remain in the system longer.

The exploration of the issue of demographic ageing provides a significant theoretical and practical contribution to the issue of ensuring adequate staffing levels in the healthcare service. Forming an understanding of the dynamics of this process and uncovering new factors that influence it have the potential to yield valuable insight for experts, policymakers and authors of strategic documents. The planning of measures in areas such as pension security, health and social services, housing, education and the promotion of intergenerational solidarity should be based on current data and trends rather than solely on historical experience.

Furthermore, this issue is particularly timely in the Czech context since many previous studies relied on outdated data and there remains a lack of research that interlinks demographic analysis with reflections on the extraordinary events of recent years, including, for instance, the impact of the Covid-19 pandemic on the health status, mortality and morbidity of seniors, labour market shifts related to the development of artificial intelligence, and the influence of migration from war-torn Ukraine on the age and social structure of the Czech population. Similarly, in the Czech environment, it is necessary to contextualise demographic ageing within the healthcare system. The ageing of healthcare personnel has, for the past 20 years, been considered one of the most fundamental themes in terms of both health policy and professional research. The average age of physicians and nurses is gradually increasing in most developed countries, which is significantly impacting the accessibility, quality and long-term sustainability of healthcare services. Research has repeatedly demonstrated that the age composition of both physicians and nurses is shifting towards older age cohorts over time.^22^

Armed with reliable data and quality research on the demographic ageing of healthcare professionals, it is possible to better anticipate and time their retirement or to adjust conditions and demands in a timely manner, thus encouraging ageing healthcare workers to remain in employment for several additional years. Moreover, it is necessary to provide support for the generational renewal of both male and female physicians in the Czech healthcare system, even though it is most likely too late to achieve full replacement in this regard. The understanding of the dynamics of ageing and the identification of new factors that influence this process have the potential to provide valuable insight for the scientific community, policymakers and strategic planners. Thus, the study of the demographic ageing of healthcare professionals provides the ideal foundation for both theoretical and practical advancement in this area.

## Data Availability

The data that supports the findings of this study are available from the General Health Insurance Company (GHIC); however, restrictions apply to the availability of this data, which was used for the purposes of this study under contract, and is, therefore, not publicly available. Nevertheless, the data is available from the authors upon reasonable request and with the consent of the GHIC.*
